# Performance of hospitals according to the ESC ACCA quality indicators and 30-day mortality for acute myocardial infarction: national cohort study using the United Kingdom Myocardial Ischaemia National Audit Project (MINAP) register

**DOI:** 10.1093/eurheartj/ehx008

**Published:** 2017-02-20

**Authors:** Owen Bebb, Marlous Hall, Keith A. A. Fox, Tatendashe B. Dondo, Adam Timmis, Hector Bueno, François Schiele, Chris P. Gale

**Affiliations:** 1 Leeds Institute of Cardiovascular and Metabolic Medicine, University of Leeds, Leeds, UK; 2 York Teaching Hospital NHS Foundation Trust, York, UK; 3 Centre for Cardiovascular Science, University of Edinburgh, UK; 4 NIHR Cardiovascular Biomedical Research Unit, Barts Heart Centre, London; 5 Centro Nacional de Investigaciones Cardiovasculares (CNIC), Spain; 6 Instituto de investigación and Cardiology Department, Hospital Universitario, Spain; 7 Universidad Complutense de Madrid, Spain; 8 Department of Cardiology, University Hospital Jean Minjoz, Besancon, France

**Keywords:** Quality indicators, Acute myocardial infarction, Mortality, Hospital performance

## Abstract

**Aims:**

To investigate the application of the European Society of Cardiology Acute Cardiovascular Care Association quality indicators (QI) for acute myocardial infarction for the study of hospital performance and 30-day mortality.

**Methods and results:**

National cohort study (n = 118,075 patients, n = 211 hospitals, MINAP registry), 2012-13. Overall, 16 of the 20 QIs could be calculated. Eleven QIs had a significant inverse association with GRACE risk adjusted 30-day mortality (all P < 0.005). The association with the greatest magnitude was high attainment of the composite opportunity-based QI (80-100%) vs. zero attainment (odds ratio 0.04, 95% confidence interval 0.04-0.05, P < 0.001), increasing attainment from low (0.42, 0.37- 0.49, P < 0.001) to intermediate (0.15, 0.13-0.16, P < 0.001) was significantly associated with a reduced risk of 30-day mortality. A 1% increase in attainment of this QI was associated with a 3% reduction in 30-day mortality (0.97, 0.97-0.97, P < 0.001). The QI with the widest hospital variation was ′fondaparinux received among NSTEMI′ (interquartile range 84.7%) and least variation ′centre organisation′ (0.0%), with seven QIs depicting minimal variation (<11%). GRACE risk score adjusted 30-day mortality varied by hospital (median 6.7%, interquartile range 5.4-7.9%).

**Conclusions:**

Eleven QIs were significantly inversely associated with 30-day mortality. Increasing patient attainment of the composite quality indicator was the most powerful predictor; a 1% increase in attainment represented a 3% decrease in 30-day standardised mortality. The ESC QIs for acute myocardial infarction are applicable in a large health system and have the potential to improve care and reduce unwarranted variation in death from acute myocardial infarction.

## Introduction

Between and within European country variation in the delivery and outcomes from acute myocardial infarction [AMI] suggest that the potential to reduce the burden of cardiovascular disease has not been realized.^1–3^ Measuring recognized standards of care is a mechanism by which geographic variation in the use of guideline-indicated treatments may be addressed and, therefore, cardiovascular outcomes improved. The 2016 European Society of Cardiology [ESC] Acute Cardiovascular Care Association [ACCA] quality indicators [QI] for the management of AMI^4^ are based upon the ESC guidelines for the management of AMI in patients presenting with ST-segment elevation^5^ and acute coronary syndrome in patients presenting without persistent ST-segment elevation.^6^ They comprise 7 domains across 20 QIs, including the evaluation of: (1) centre organization, (2) the reperfusion/invasive strategy, (3) in hospital risk assessment, (4) antithrombotic treatment during hospitalization, (5) secondary prevention discharge treatments, (6) patient satisfaction, and (7) composite QIs and Global Registry of Acute Coronary Events (GRACE) risk score adjusted 30-day mortality.

To date, there has been no investigation of within country provider variation according to the ESC ACCA QIs or the relationship between the QIs and 30-day mortality. To address this knowledge gap, providing an external validation of the ESC ACCA QIs for AMI, we used data from the United Kingdom national heart attack register (Myocardial Ischaemia National Audit Project [MINAP]) which collects data from one health system, the National Health Service of England and Wales.

## Methods

### Setting and design

The analyses were based on data from MINAP, a comprehensive registry of ACS hospitalizations mandated by the United Kingdom Department of Health.^7^ Each MINAP entry provides patient demographic data and clinical details of the patient journey across 122 data items; details of MINAP data collection and management have been described previously.^7^ The analytical cohort (*n* = 118 075) was drawn from patients (*n* = 118 168) with a discharge diagnosis of AMI admitted to one of 220 hospitals between 1st January 2012 and 30th June 2013 ( see Supplementary material online, *Figure S1*). Patients were eligible for the study if they were ≥18 years of age. For patients with multiple admissions the earliest record was used (to reduce potential bias from previous treatments). We excluded nine hospitals that treated ≤30 patients within the 18-month period of study. Patient-level data concerning demographics, cardiovascular risk factors, medical history, and clinical characteristics at the time of hospitalization were extracted from the registry. Unique patient identifiers were used to link patients with the Office for National Statistics such that vital status or date of death at 30 days could be ascertained. Data used within the study were fully anonymized and, as such, ethical approval was not required under NHS research governance arrangements. The National Institute for Cardiovascular Outcomes Research (NICOR) which includes the MINAP database (Ref: NIGB: ECC 1-06 (d)/2011) had support, under section 251 of the National Health Service Act 2006, to use patient information for medical research without consent. The study was conducted in compliance with the Declaration of Helsinki.

### ESC quality indicators

The ESC ACCA position statement defined 7 domains of care encompassing 12 main and 8 secondary QIs (see Supplementary material online, *Figure S2*). All 20 QIs were mapped to each patient’s MINAP data to identify data fields that would enable their calculation. For each QI, we included patients who were eligible for the treatment or intervention and whose record had no missing data. As such, patients were classified as ineligible if a treatment was contra-indicated, not indicated, not applicable, if the patient declined treatment or treatment was deemed inappropriate due to co-morbidity.

Domain seven specifies the use of an opportunity-based composite score and an all-or-none score (see Supplementary material online, *Appendix S1*). For this study, we calculated the composite score for each patient and subsequently each hospital, based on the number of times particular care processes were performed (numerator) divided by the number of chances a patient had to receive/hospital had to provide that care (denominator). The composite score was calculated using an equal weight method and included 9 measures (see Supplementary material online, *Appendix S1*).

### Statistical analyses

Baseline characteristics for the study population were described using numbers and percentages for categorical data, and medians and IQR or means and standard deviations (SD) for continuous non-normally and normally distributed data. For the QIs, the proportion presented is of those eligible for treatment.

We used a validated method for use with MINAP data to calculate each patient’s GRACE risk score. This used the scoring system and coefficients described by the GRACE investigators, given that not all records had information about Killip class and chronic renal failure, the ‘use of loop diuretic’ (as a surrogate for Killip class II-IV), and creatinine concentration, respectively, were added to each patient’s score.^8^^,^^9^

To estimate the GRACE risk score adjusted 30-day mortality, we used the predicted probabilities derived from a logistic regression model where the dependent variable was 30-day mortality and the independent variable was each patient’s calculated GRACE risk score. Data were summarized overall and at the hospital level. We used Spearman’s rank test to investigate the relationship between all combinations of QIs, except for the composite scores because they incorporated several single QIs. We fitted a logistic regression model to assess the strength of association between QI measures and 30-day mortality. For the composite opportunity measure, the performance was split into 4 categories: (1) no interventions received, (2) <40% of eligible interventions received, (3) ≥40% to <80% of eligible intervention received, and (4) ≥80% of interventions received.^10^^,^^11^ We excluded measures that had ≤30 patients with complete data for either aspect of the QI. All analyses were conducted using Stata MP Version 14.0 (StataCorp LP, TX, USA), with statistical significance determined at 5%.

## Results

### Patient characteristics

**Figure 1:**
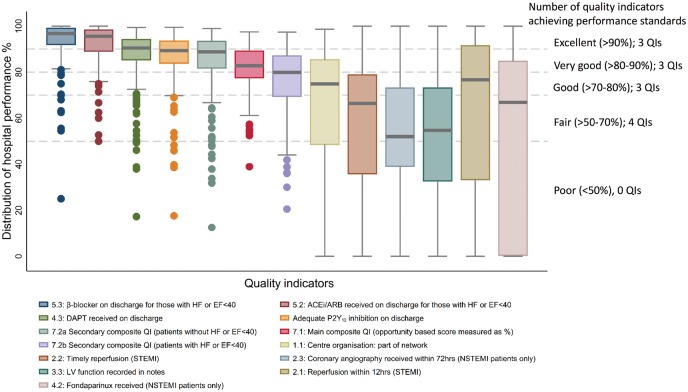
Distribution of hospitals’ performance according to the European Society Cardiology; Acute Cardiovascular Care Association quality indicators for acute myocardial infarction.

Across 211 hospitals in England and Wales, (47 341 [40.1%] STEMI and 70 734 [59.9%] NSTEMI; mean age 68.5 (SD 14.0) years; 33.2% female), there were 37 487 (34.2%) patients with a history of ischaemic heart disease, 24 068 (21.2%) with diabetes, 5,579 (5.1%) with a history of heart failure, and 6678 (6.2%) with chronic renal failure (*Table 1*). The mean GRACE score was 119.8 (SD 34.1). Following hospitalization, 83 740 (78.2%) received coronary angiography and 2605 (2.5%) coronary artery bypass grafting [CABG]. Of STEMI, 21 567 (56.7%) received primary percutaneous coronary intervention [PCI] and of NSTEMI, 23 172 (40.2%) received sub-acute or elective PCI. Hospital variation in patients’ characteristics was small, compared with wider variation in QI attainment (*Tables 1* and *2*, *Figure 1*). At 30 days, there were 7063 (7.1%) deaths.

**Table 1:** Baseline characteristics and their hospital variation of patients with acute myocardial infarction, MINAP 2012–13

	Total cohort (*n* = 118 075)	Missing data, *n*, (%)	Hospital variation, mean (SD) or median (IQR) (*n* = 211 hospitals)
Demographics			
Age in years, mean (SD)	68.5 (14.0)	63 (0.05)	69.5 (3.4)
Female	39 088 (33.2)	352 (0.30)	34.9 (30.4–38.6)
Medical history			
History of ischaemic heart disease	37 487 (34.2)	8491 (7.2)	36.1 (29.4–40.9)
Hypertension	55 397 (50.6)	8522 (7.2)	49.7 (44.1–56.0)
Diabetes	24 068 (21.2)	4730 (4.01)	21.6 (17.7–24.4)
Dyslipidaemia	36 890 (34.2)	10 296 (8.72)	30.4 (22.6–40.6)
Family history of ischaemic heart disease	28 936 (30.5)	23 281 (19.7)	22.1 (16.3–30.8)
Smoker (current or previous)	67 670 (61.4)	7933 (6.7)	57.7 (50.4–63.5)
Peripheral vascular disease	4699 (4.3)	9659 (8.2)	3.9 (2.6–5.3)
Congestive cardiac failure	5579 (5.1)	9247 (7.8)	5.3 (2.93–8)
COPD	16 326 (15.0)	9323 (7.9)	15.1 (12.1–17.6)
Chronic kidney disease	6678 (6.2)	9854 (8.4)	6.1 (3.4–8.7)
Cerebrovascular disease	9070 (8.4)	9489 (8.0)	8.2 (5.7–10.9)
Clinical Presentation			
Heart rate at hospitalization, median (IQR), b.p.m.	78 (66–91)	18 887 (16.0)	78 (76–80)
Systolic blood pressure at hospitalization, mean (SD), mmHg	136 (27.8)	18 794 (15.9)	139 (5.2)
Out of hospital cardiac arrest	3287 (2.9)	3737 (3.2)	1.9 (0.6–3.3)
Initial creatinine, median (IQR), µmol/L	86 (72–107)	11 622 (9.8)	87 (83–90)
ST-segment deviation on admission	61 439 (53.5)	3140 (2.7)	38.8 (25.8–57.3)
Killip class^c^		36 310 (30.8)	
I	64 254 (78.6)		78.1 (70.6–86.6)
II	11 697 (14.3)		14.1% (6.2–21.0)
III	4424 (5.4)		5.0 (3.1–8.0)
IV	1390 (1.7)		0.6 (0–1.7)
GRACE risk score, mean (SD)	119.8 (34.1)	33 536 (28.4)	119.6 (114.3–123.7)
In hospital revascularization, (of those eligible)			
Angiogram^b^	83 740 (78.2)	4210 (3.6)	67.5 (52.7–85.0)
PCI	67 740 (65.6)	15 388 (13.0)	45.9 (28.0–73.7)
CABG	2605 (2.5)	15 388 (13.0)	1.2 (0.2–3.5)
Medications at discharge,^d^			
Aspirin	101 591 (98.1)	1374 (1.2)	98.7 (96.7–99.6)
P2Y_12_ inhibitor	92 501 (87.1)	1434 (1.2)	89.3 (83.9–93.5)
β-blocker	86 543 (95.6)	1412 (1.3)	96.5 (92.8–98.8)
Statin	84 421 (96.5)	1275 (1.14)	96.9 (93.1–98.9)
ACEi/ARB	84 681 (93.9)	1480 (1.33)	94.6 (89.5–98.2)
Lifestyle advice,			
Cardiac rehabilitation	88 302 (81.7)	4340 (3.7)	85.5 (70.5–94.9)
Smoking cessation advice	27 848 (74.4)	2222 (3.3)	78.0 (54.7–90.5)
Dietary advice	81 745 (77.4)	7484 (6.3)	86.5 (55.3–95.9)

Values presented are given as number  (percentage) unless stated.

SD, standard deviation; IQR, interquartile range; IHD, ischaemic heart disease; COPD, chronic obstructive pulmonary disease; GRACE, Global Registry Acute Coronary Events; ACEi/ARB, angiotensin converting enzyme inhibitor/angiotensin II receptor blocker; PCI, percutaneous coronary intervention; CABG, coronary artery bypass grafting.

a History of ischaemic heart disease refers to a history of CABG, MI, PCI, or angina.

b Received angiography or PCI.

c Killip class; 1: No clinical signs of heart failure, 2: Rales/crackles within the lungs, present S_3_, elevated JVP, 3: frank pulmonary oedema, 4: cardiogenic shock.

d Includes eligible patients who started mediations during admission.

**Table 2:** Overall and hospital variation in performance according to the European Society Cardiology; Acute Cardiovascular Care Association quality indicators for acute myocardial infarction

Domain	Quality indicator	Type of quality indicator	Total patients eligible (*n*)	Proportion receiving care (%)	Hospital variation median % (IQR) (*n* = 211 hospitals)
1: Centre organization	1.1 Centre organization: part of network	Main	76 099	77.8	74.9 (48.6–85.3)
	1.1a: Single emergency phone number to allow medical triage		118 075	100	100 (100–100)
	1.1b: Pre hospital interpretation of the ECG		76 099	77.8	74.9 (48.6–85.3)
	1.1c: Pre hospital activation of the catheter lab		118 075	100	100 (100–100)
	1.2: Routine assessment of times to reperfusion	Secondary	118 075	100	100 (100–100)
	1.3: Participate in regular registry	Secondary		100	100 (100–100)
2: Reperfusion/invasive strategy	2.1: Reperfusion within 12 h of presentation (STEMI)	Main	33 151	89.3	76.7 (33.3–91.4)
	2.2 Timely reperfusion (STEMI)	Main	27 892	74.6	66.4 (35.8–78.8)
	2.2a: Fibrinolysis received within 30 min (PPCI centres and STEMI patients only)		547	55.0	33.3 (0–60.6)
	2.2b: Primary PCI received within 60 min (PPCI centres and STEMI patients only)		26 358	75.0	69.9 (54.6–80.8)
	2.2c: Primary PCI received within 120 min (non-PPCI centres and STEMI patients only)		672	93.9	40.0 (0–53.3)
	2.2d: Door-in door-out time within 30 min (non-PPCI centres and STEMI patients only)		538	23.8	5.7 (0–49.6)
	2.3: Coronary angiography received within 72 h (NSTEMI patients only).	Main	29 199	61.3	52.0 (39.1–73.2)
	2.4: Time from diagnosis to wire passage (STEMI), minutes (median, IQR)	Secondary	27 029	185 (135–284)	187.8 (169.8–210)
3: In Hospital risk assessment	3.1: GRACE risk score recorded in notes	Main	N/A	0	N/A
	3.2: CRUSADE risk score recorded in notes	Main	N/A	0	N/A
	3.3: LV function recorded in notes	Main	104 004	54.5	54.7 (32.7–73.2)
4: Anti thrombotics during hospital	4.1: Adequate P2Y_12_ inhibition on discharge	Main	106 157	87.1	89.3 (83.8–93.5)
	4.2: Fondaparinux received (NSTEMI patients only)	Main	61 152	50.3	66.8 (0.4–84.7)
	Fondaparinux or LMWH received (NSTEMI patients only)		61 185	85.2	90.3 (84.2–94.6)
	4.3: DAPT received on discharge	Secondary	101 582	88.1	90.5 (85.4–94.1)
5: Secondary prevention	5.1: High intensity statins on discharge	Main	N/A	0	N/A
	5.2: ACEi/ARB on discharge for those with HF or LVEF ≤40	Secondary	33 531	94.2	95.5 (89.1–98.4)
	5.3: β-blocker on discharge for those with HF or LVEF ≤40	Secondary	34 150	95.8	96.8 (92.0–99.0)
6: Patient satisfaction	6.1 Not applicable	Main	N/A	N/A	N/A
7: Composite QI	7.1 Composite QI (opportunity-based)	Main	118 071	83.3 (75.0–100)	82.8 (77.6–89.1)
	7.2 Composite QI (all-or-none, overall score)	Secondary	118 075	83.1	84.8 (76.7–90.5)
	7.2a Composite QI (all-or-none, 3 measures) (%) patients with no HF or LVEF≤40	Secondary	72 648	84.8	88.8 (81.8–93.3)
	7.2b Composite QI (all-or-none, 5 measures) (%) patients with HF or LVEF≤40	Secondary	45 427	80.2	79.9 (69.5–87.0)
	7.3 Mortality rate adjusted for GRACE risk		84 539	6.9 S.D 10.4	6.7 (5.4–7.9)

Abbreviations: IQR, interquartile range; ECG, electrocardiogram; STEMI, ST-elevation myocardial infarction; PPCI, primary percutaneous coronary intervention; PCI, percutaneous coronary intervention; NSTEMI, non-ST elevation myocardial infarction; GRACE, Global Registry Acute Coronary Events; CRUSADE, Can Rapid Risk Stratification of Unstable Angina Patients Suppress ADverse Outcomes with Early Implementation of the ACC/AHA Guidelines); LV, left ventricular; LMWH, low molecular weight heparin; eGFR, estimated glomerular filtration rate; DAPT, dual antiplatelet therapy; ACEi/ARB, angiotensin converting enzyme inhibitor/angiotensin II receptor blocker; QI, quality indicator; HF, heart failure; LVEF, left ventricular ejection fraction; N/A, not applicable.

### Domains and quality indicators

Of the 7 QI domains, MINAP contained data fields for the assessment of all, except the evaluation of patient satisfaction (*Table 2*). MINAP allowed the assessment of care according to 16 of the 20 QIs; 12 derived directly from corresponding data fields and 4 ascertained indirectly. The remaining 4 quality indicators including, the prescription of high intensity statins at hospital discharge, the recording of the GRACE and CRUSADE risk scores, and patient satisfaction could not be evaluated because MINAP did not collect this information. *Figure 1* demonstrates the attainment and variation at a centre level for those QIs measured.

*Domain 1: Centre* *organization* *.* Overall, 77.8% (*n* = 76 099) of eligible patients had pre-hospital interpretation of an ECG, higher than the median value for hospitals 74.8% (IQR 48.6–85.3%). For the remaining components of the main QI and both of the secondary QIs, the level of attainment for patients with AMI was 100% (*n* = 118 075) (*Table 2*).

*Domain 2: Reperfusion/invasive strategy.* For STEMI, 89.3% (*n* = 33 151) received reperfusion <12 h of onset of symptoms, and 74.6% (*n* = 20 815) received timely reperfusion. (*Table 2*) The median time from first medical contact with STEMI to wire passage for the whole cohort was 185 (IQR 135–284) min which was similar to the median time for the hospitals, although variation was less (188 min, IQR 170–210 min). For NSTEMI, the performance of coronary angiography was low and varied between hospitals (median, 52.0%, IQR 39.1–73.2%), and 61.3% (*n* = 17 895) received coronary angiography <72 h of hospitalization.

*Domain 3: In hospital risk assessment.* Only one of the main QIs could be assessed; the assessment of LVEF was recorded in 54.5% (*n* = 56 680) of eligible patients, and demonstrated suboptimal attainment which varied by hospital (median 54.5%, IQR 32.7–73.2%).

*Domain 4: Antithrombotic treatment during* *hospitalization* *.* The prescription of adequate P2Y_12_ inhibition at discharge was achieved in 87.1% (*n* = 92 501), but varied across hospitals (median 89.3%, IQR 83.8–93.5). Fondaparinux use was low (50.3%, *n* = 30 737) and exhibited the greatest hospital variation (median 66.8%, IQR 0.4–84.7%). However, when fondaparinux or low molecular weight heparin was considered, performance improved and variation reduced (median 90.3%, IQR 84.2–94.6%). The secondary QI found that 88.1% (*n* = 89 488) of eligible patients with AMI were discharged on dual antiplatelet therapy.

*Domain 5: Secondary prevention discharge treatments.* In total, 96.5% (*n* = 84 421) of patients eligible for lipid lowering therapy were prescribed a statin at time of discharge from hospital. For the two secondary QIs, 94.2% (*n* = 31 569) of patients with AMI and heart failure or a LVEF ≤0.40 received an ACEi or ARB, and 95.8% (*n* = 32 728) with AMI and heart failure or a LVEF ≤0.40 received a β-blocker. Hospital attainment was high, but varied between hospitals (IQR 89.1–98.4 and 92.0–99.0, respectively).

*Domain 6: Patient Satisfaction.* The MINAP registry recorded no data about patient satisfaction during the period of study. However, 81.7% (*n* = 108 110) of patients were referred for cardiac rehabilitation, 77.4% (*n* = 105 603) received dietary advice and 74.4% (*n* = 37 443) of current smokers received cessation advice. Across hospitals 85.5% of patients were referred for cardiac rehabilitation (IQR 70.5–94.9), 86.5% of smokers received cessation advice (IQR 54.7–90.5%), and 86.5% were offered dietary advice (IQR 55.3–95.9%).

**Figure 2:**
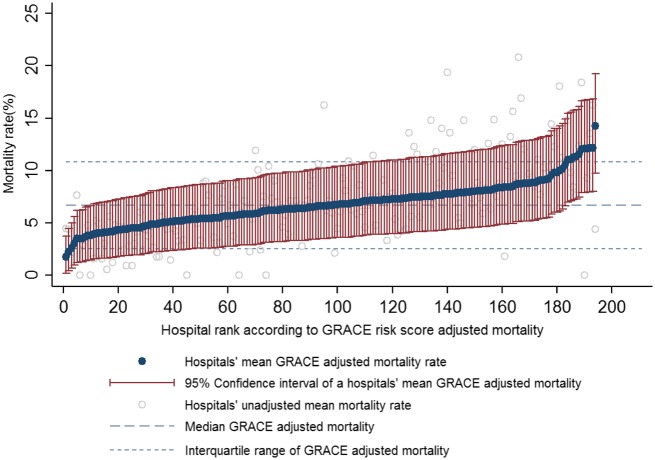
Caterpillar plot of hospital rank of hospital mean unadjusted and mean GRACE risk score adjusted hospital 30-day mortality rates.

*Domain 7: Composite quality indicators and GRACE risk score adjusted 30-day mortality.* According to the opportunity-based composite score, patients received 83.3% (*n* = 118 071) of the interventions for which they were eligible. Hospital attainment was high, but varied between hospitals (median of 82.8%, IQR 77.5–88.7%). For the all-or-none composite score, 83.1% of patients received all of the interventions for which they were eligible which varied more among patients with heart failure or an ejection fraction ≤0.40 than those without (IQR 69.5–87.0 vs. 81.8–93.3). For the cohort, the median GRACE risk score adjusted 30-day mortality was 2.7% (IQR 0.9–8.1%). At the hospital level, variation was limited (median 6.7%, IQR 5.4–7.9%) (*Figure 2*).

### Correlation of quality indicators

**Figure 3:**
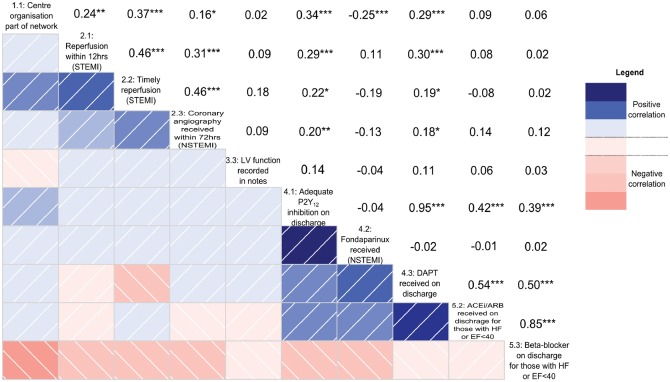
Scatter matrix of European Society Cardiology; Acute Cardiovascular Care Association quality indicators for acute myocardial infarction showing pairwise correlations of all quality indicator pairs, presented alongside Spearman’s rank correlation coefficient (where * indicates *P* < 0.05, ** *P* < 0.01, ***P < 0.001).

Overall 39 of the 45 QI to QI combinations demonstrated a weak correlation (Spearman correlation coefficient <0.3), 4 had a significant moderate correlation (0.4–0.7, all *P* < 0.001) and 2 had a significant strong correlation (≥0.7, all *P* < 0.001) (*Figure 3*).

### Quality indicators and mortality

**Figure 4:**
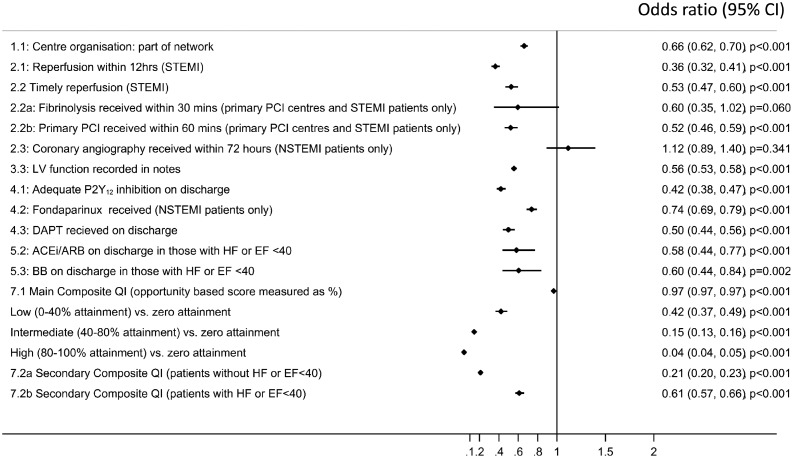
Association between the European Society Cardiology; Acute Cardiovascular Care Association quality indicators for acute myocardial infarction and crude 30-day mortality. The composite opportunity QI was divided into the following categories: zero–received no interventions out of those eligible for, low–received <40% of interventions eligible for, intermediate–received ≥40 to <80% of interventions eligible for and high–received ≥80% of interventions eligible for.

Eleven QIs had a significant inverse association with 30-day mortality (all *P* < 0.005) (*Figure 4*). The association with the greatest magnitude was for high attainment vs. zero attainment of the composite opportunity-based QI (odds ratio [OR] 0.04, 95% confidence interval [CI] 0.04–0.05, *P* < 0.001). Increasing patient attainment of this indicator from low (OR 0.42 CI 0.37–0.49, *P* < 0.001) to intermediate (0.15, 0.13–0.16, *P* < 0.001) to high (0.05, 0.04–0.06, *P* < 0.001) was significantly associated with a lower risk of 30-day mortality. On average, a unit increase in percentage attainment was significantly associated with a 3% decrease in 30-day mortality (0.97, 0.97–0.97, *P *<* *0.001).

## Discussion

Using a nationwide clinical database, comprising an analytical cohort of nearly 120 000 patients and over 200 hospitals between 2012–13, we found that the ESC ACCA QIs for AMI allowed the thorough evaluation of AMI care against international standards. The majority of QIs assessed were significantly inversely associated with 30-day mortality, the strongest being a composite indicator which, with increasing attainment, was associated with decreasing rates of death in a dose-response manner. Whilst we found high levels of performance with associated low levels of mortality, there was evidence for between hospital variation in key metrics, which mapped to class 1 guideline-indicated care. As such, the ESC QIs for AMI are applicable and valid, highlighting where in health systems there is potential to improve care and that high levels of performance according to the QIs is likely to reduce unwarranted variation and premature death from AMI.

Data from the EUROASPIRE studies show that the use of evidence-based treatments for AMI and associated outcomes vary widely between European countries.^12–14^ Other international comparisons provide evidence for variation both between and within countries.^1^^,^^3^^,^^15–17^ When there are data to show that adherence to guidelines improves clinical outcomes,^3^^,^^18^ variation in healthcare performance against set standards serves as indirect evidence for the potential to modify mortality. Results from our study show that whilst there was variation between hospitals in baseline patient characteristics, qualitatively this was less that that derived for the QIs, suggesting that the provision of treatments may have a greater role in determining outcomes than case mix.^19^

The association between risk adjusted mortality and the ESC ACCA QIs is consistent with previous findings.^20^^,^^21^ However, this study evaluates a wider spectrum of QIs which map to guidelines and transcend the pathway of AMI care as well as organization of services. Four of the QIs were not associated with a significant reduction in mortality. Coronary angiography <72 h for patients with NSTEMI demonstrated a positive association with mortality. When further investigated, however, the provision of PCI was inversely associated with mortality (OR 0.58, 95% CI 0.51–0.65), in keeping with other evidence.^19^

We noted many QIs did not correlate with each other, implying they cover the spectrum of the AMI care pathway. The greatest correlation was between the prescription of P2Y_12_ inhibitors and dual antiplatelet therapy–given that the former is essential for the latter, this is not unexpected. The weakest association was for centre organization and timely angiography in NSTEMI; given that in the United Kingdom centre organization was primarily arranged to treat STEMI, it is not surprising that these two measures did not correlate.

Variation in the delivery of treatments is dependent upon many factors, including the availability of sufficient hospital facilities,^16^^,^^22^ a skilled workforce,^15^^,^^22^ high levels of knowledge transfer from scientific studies between healthcare professionals,^23^ the volume of cases admitted to the hospital,^24^ differences in the extent to which care is felt to be appropriate,^25^ as well as uniformity of recommendations from guidelines from different countries.^17^ Regarding the latter, we noted that the QI with the widest hospital variation was that for fondaparinux. We speculate this may be because the United Kingdom (2010) guideline for the management of patients with AMI,^26^ recommended fondaparinux only for cases of AMI who were not going for angiography <24 h of hospitalization–therein differing from current ESC recommendations. The QI with the least variation was centre organization. This is because, in the United Kingdom, emergency care for STEMI is institutionally operationalized as a result of the implementation of a national primary PCI service.^22^

In North America, there is a well-established program of quality improvement that, for AMI is based upon the ACTION registry^27^ and allows benchmarking of performance comparisons of providers. For the European community, the ESC AMI QIs offer an opportunity to study and consequently address deficits in care for cardiovascular disease. We demonstrate that this is possible only through a comprehensive clinical registry, as have others,^27^ but which for several European countries is lacking.^1^

Although this study has many strengths, we recognize its limitations. The findings are summary measures of performance grounded on patient-level data and described at a hospital level. We followed the ESC AMI QI specification for the calculation of adjusted mortality,^4^ being mindful that hospital-specific influences were not accounted for in the modelling. Although we excluded hospitals with ≤30 patients recorded during the study period, for some hospitals in the separate QI analytical cohorts had ≤30 patients. For the GRACE score, we used surrogates for both Killip class and creatinine in keeping with previous validation work.^8^^,^^9^ MINAP does not record the specific type of statins, so ‘statin prescription’ was used as a surrogate for high intensity statin. Similarly, because there was imperfect recording of Ticagrelor, we used instead receipt of P2Y_12_ inhibitor.

The recoding, measurement, and reporting of within and between country performance against validated QIs representing class 1 indicated care is the critical next step in the international effort to reduce the burden and variation in premature deaths due to cardiovascular disease across Europe. This study provides good evidence for the application of the ESC ACCA QIs for AMI to clinical registries for the evaluation of cardiovascular care and outcomes; demonstrating their significant inverse association with mortality. Furthermore, this study identified potentially modifiable variation within a high performing health system and sets a road map for the development of standardized data collection in other ESC member countries. Greater and more uniform adherence to guideline-indicated care will result in improved and less varied mortality from AMI.

## Supplementary material

Supplementary material is available at *European Heart Journal* online.

## Supplementary Material

Supplementary Data
