# Supplementary material for: Performance of hospitals according to the ESC ACCA quality indicators and 30-day mortality for acute myocardial infarction: national cohort study using the United Kingdom Myocardial Ischaemia National Audit Project (MINAP) register
Source: Eur Heart J. 2017 Feb 20;38(13):974–82. doi: 10.1093/eurheartj/ehx008 (PMC5724351; doi:10.1093/eurheartj/ehx008)
Supplement: Supplementary Data [file ehx008_supp.zip › supp fig 3 forrest plot adjusted mortality.docx]

**Supplementary figure 3:** Association between the European Society Cardiology; Acute Cardiovascular Care Association quality indicators for acute myocardial infarction and GRACE risk adjusted 30-day mortality*


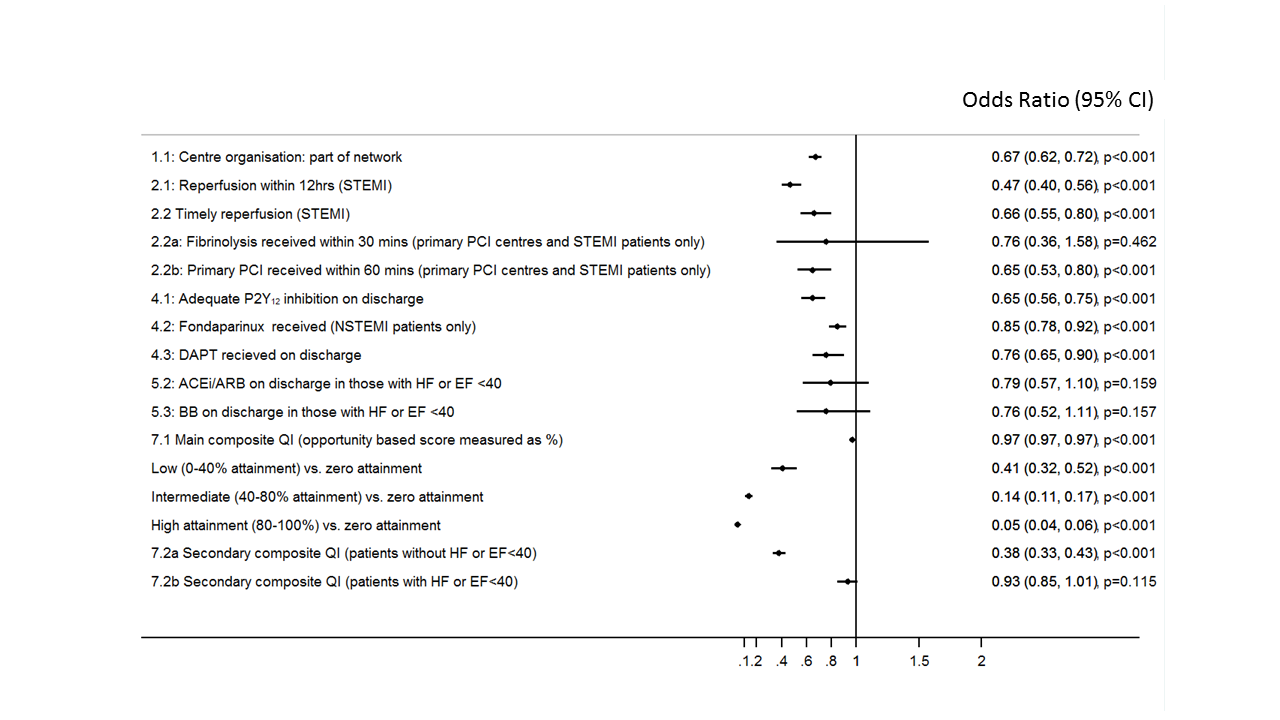


*Logistic regression of mortality in patients with a GRACE score for the QI and GRACE risk score

**Key**

STEMI: ST-segment elevation myocardial infarction, PCI: percutaneous coronary intervention, NSTEMI: non-ST elevation myocardial infarction, LV: left ventricular, DAPT; dual antiplatelet therapy, ACEi/ARB: angiotensin converting enzyme inhibitor / angiotensin receptor blocker, HF: heart failure, EF: ejection fraction, BB: β blocker, QI: quality indicator

**Explanation**

The composite opportunity QI was divided into the following categories: zero - received no interventions out of those eligible for, low - received up to 40% of interventions eligible for, intermediate - received between 40 and 80% of interventions eligible for and high - received >80% of interventions eligible for.
