# Supplementary material for: Performance of hospitals according to the ESC ACCA quality indicators and 30-day mortality for acute myocardial infarction: national cohort study using the United Kingdom Myocardial Ischaemia National Audit Project (MINAP) register
Source: Eur Heart J. 2017 Feb 20;38(13):974–82. doi: 10.1093/eurheartj/ehx008 (PMC5724351; doi:10.1093/eurheartj/ehx008)

**Supplementary Figure 1:** Schematic representing the ESC ACCA QIs for AMI domains and their components


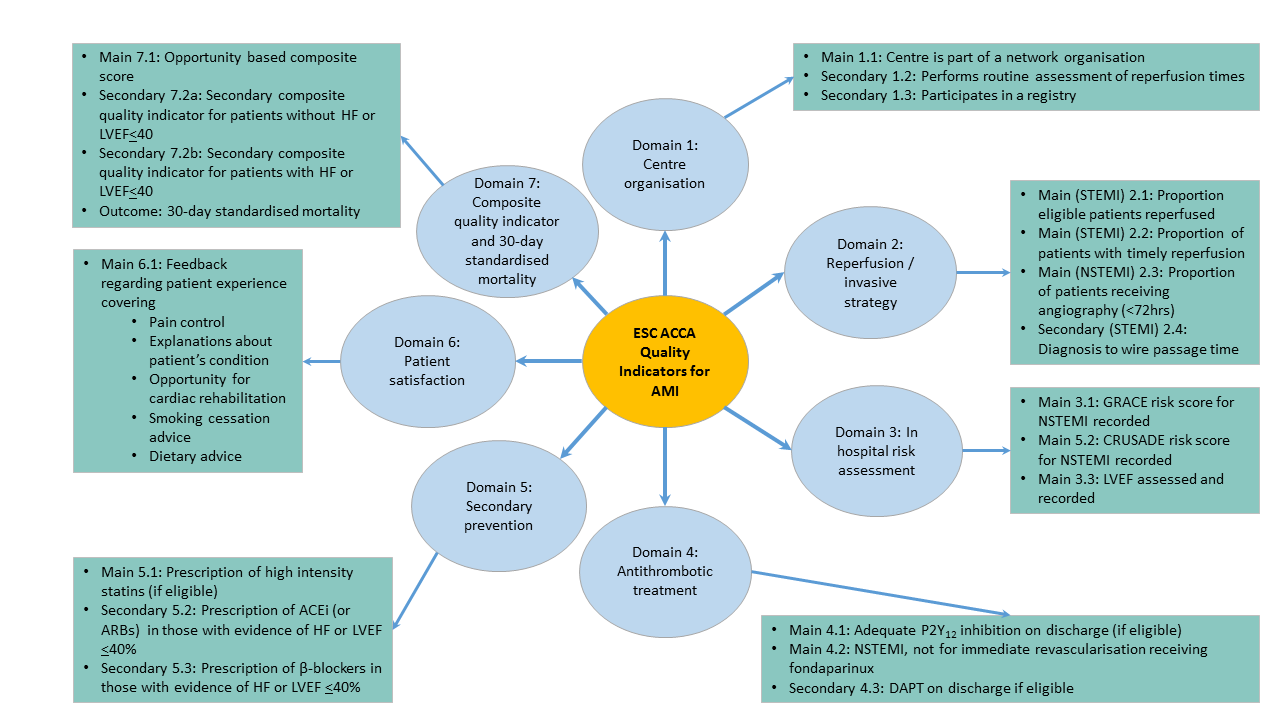

Supplement: Supplementary Data [file ehx008_supp.zip › Supplementary figure 1 26.10.docx]
