# Supplementary material for: Performance of hospitals according to the ESC ACCA quality indicators and 30-day mortality for acute myocardial infarction: national cohort study using the United Kingdom Myocardial Ischaemia National Audit Project (MINAP) register
Source: Eur Heart J. 2017 Feb 20;38(13):974–82. doi: 10.1093/eurheartj/ehx008 (PMC5724351; doi:10.1093/eurheartj/ehx008)
Supplement: Supplementary Data [file ehx008_supp.zip › Supplementary Figure 2 STROBE Diagram.docx]

Supplementary Figure 2: STROBE Diagram; demonstrating ascertainment of final investigative cohort

733,863 STEMI/NSTEMI

243Hospitals

First admission patients recorded in MINAP

 Exclusion Criteria

All admissions 2003-2010

(n=615695 patients)

(23 hospitals)

118,168 STEMI/NSTEMI

220 Hospitals

First admission patients in 2012/2013

Final Cohort of Patients

118,075 STEMI/NSTEMI

211 Hospitals

 Exclusion Criteria

All hospitals with < 30 patients in that time frame

(n=93 patients) (9 Hospitals)

Key:

MINAP; myocardial national ischaemia audit project, STEMI; ST-elevation myocardial infarction, NSTEMI; non-ST-elevation myocardial infarction
